# Tablet Geometry Effect on the Drug Release Profile from a Hydrogel-Based Drug Delivery System

**DOI:** 10.3390/pharmaceutics15071917

**Published:** 2023-07-10

**Authors:** Seyed-Farid Mohseni-Motlagh, Roshanak Dolatabadi, Majid Baniassadi, Morad Karimpour, Mostafa Baghani

**Affiliations:** 1 School of Mechanical Engineering, College of Engineering, University of Tehran, Tehran 1439957131, Iran; 2 Department of Drug and Food Control, Faculty of Pharmacy, Tehran University of Medical Sciences, Tehran 1416634793, Iran; 3 Food and Drug Administration, Iran Ministry of Health and Medical Education, Tehran 1419943471, Iran

**Keywords:** QbD, release profile, hydrogel, HPMC, drug delivery, mathematical modeling, geometry, MDT, AUC, DE

## Abstract

In order to achieve the optimal level of effectiveness and safety of drugs, it is necessary to control the drug release rate. Therefore, it is important to discover the factors affecting release profile from a drug delivery system. Geometry is one of these effective factors for a tablet-shaped drug delivery system. In this study, an attempt has been made to answer a general question of how the geometry of a tablet can affect the drug release profile. For this purpose, the drug release process of theophylline from two hundred HPMC-based tablets, which are categorized into eight groups of common geometries in the production of oral tablets, was simulated using finite element analysis. The analysis of the results of these simulations was carried out using statistical methods including partial least squares regression and ANOVA tests. The results showed that it is possible to predict the drug release profile by knowing the geometry type and dimensions of a tablet without performing numerous dissolution tests. Another result was that, although in many previous studies the difference in the drug release profile from several tablets with different geometries was interpreted only by variables related to the surface, the results showed that regardless of the type of geometry and its dimensions, it is not possible to have an accurate prediction of the drug release profile. Also, the results showed that without any change in the dose of the drug and the ingredients of the tablet and only because of the difference in geometry type, the tablets significantly differ in release profile. This occurred in such a way that, for example, the release time of the entire drug mass from two tablets with the same mass and materials but different geometries can be different by about seven times.

## 1. Introduction

To improve the effectiveness and safety of drug use and issues such as bioavailability or fluctuations in the level of drug concentration in the blood, it is important to control the drug release rate [1,2]. Therefore, it is necessary to provide a mechanism to release the drug from a drug delivery system at a controlled and optimal rate. Controlled drug delivery systems can cause desirable effects such as stable drug release, reducing the number of required doses, or better adaptation of the drug to the patient’s condition [3]. One of these systems, that is of great interest, is the hydrogel-based drug delivery system [4,5,6]. Hydrogels are composed of 3D hydrophilic polymer networks that are able to absorb a large amount of water and medicinal substances. They are effective and popular carriers in drug delivery systems due to the ability to slowly release drugs through a diffusion mechanism [7,8], their physical similarity to body tissue [9], low protein absorption in the body, and the ability to be used for both hydrophilic and hydrophobic drugs [10]. The mechanism of these systems is such that, first, water penetrates into the volume of the matrix so that the hydrogel contains a large amount of water and its volume increases greatly. This volume increase can be in response to a biological stimulus. This increase in volume causes the polymer chains to open and allows the drug to slowly diffuse into the dissolution medium [11,12].

When a conventional or controlled drug delivery system is used for drug release, due to the importance of the release rate, the factors affecting the drug release rate from the used system should be investigated. This issue has received more attention in recent years due to the emphasis on the use of the concept of quality by design (QbD) by the U.S. Food and Drug Administration (FDA). With the QbD initiative, routine quality control tests, such as dissolution testing (DT) performed after the production process, can be reduced or even stopped if the influencing parameters are controlled [13]. This goal can be achieved by conducting experiments or simulating the drug release process from a drug delivery system.

Modeling the behavior of hydrogel-based systems, such as hydrogel tablets, is currently a topic of great interest because, as with all phenomena, mathematical models are a necessary step to achieve a deep understanding of the process. This work leads to targeted design and reduction of costs due to frequent tests [14]. One of the key factors in choosing the type of modeling approach is paying attention to the type of responses that are needed. For example, the approach can only provide an analysis of the mass transfer process or focus on the phenomenon’s mechanical behavior. In a general classification, the approaches to modeling the drug release from hydrogel-based systems can be divided into the following three categories: 1—the drug release fitting, 2—statistical and neural network models, 3—models with a mechanical approach [14].

In drug release fitting models, which are the most widely used modeling approach due to their simplicity, the percentage of drug released from the matrix to the total mass of the initial drug is considered as a function of time. In such modeling, modeling of the amount of drug release over time from one or more drug delivery systems using equations such as Higuchi, zero-order, Korsmeyer–Peppas, or other famous equations is usually attempted [15,16,17].

In statistical and neural network models, the prediction of some important responses of the release profile based on a series of controllable parameters during the production of drug delivery system is attempted. These responses usually include cumulative percentage of released drug, matrix swelling rate, etc. Controllable parameters during the production of drug delivery systems usually include formulation, geometry, tablet surface hardness, etc. For example, in the research carried out by Barmpalexis et al., using neural network techniques, linear regression, and higher-order regressions, they were able to accurately predict responses such as mean dissolution time, maximum solvent absorption, and several other responses based on the combination ratio of four substances in drug production [18]. Studies such as Yekpe et al.’s study with emphasis on the importance of tablet surface hardness and tablet coating weight on the drug release rate [13] and Frankiewicz and Sznitowska’s study with emphasis on the importance of production temperature and coating spray pressure on the tablet on the drug release rate [19] are studies with this type of modeling approach.

Another approach is the mechanical approach. The main building block of this type of approach is Fick’s law of diffusion. In most of the old models with a mechanical approach, the analytical and sometimes numerical solution of diffusion equations is considered according to the geometry of the problem, and in fact, only the description of the drug component has been discussed [14]. For example, Arroyo et al., to achieve an optimal composition for the release of indomethacin as an anti-inflammatory cream, solved the diffusion equations in one dimension for a curved shell and presented an analytical solution [20]. But, the models that have received the most attention from researchers today focus on other aspects of drug release from a drug delivery system. For example, the model also explains the behavior of other components in the system such as water and polymer, describes swelling and erosion phenomena, and can also describe the behavior of drug delivery systems with more complex geometries [14]. One of the main steps in the introduction and presentation of such models is shown by Siepmann et al.’s research series from 1999 to 2000 [21,22,23], especially the “sequential layer” model [23]. Although their model could explain inhomogeneous deformations of a drug delivery system, there were still some shortcomings in the model. For example, the deformation was affine despite being inhomogeneous (that is, for example, a cylindrical system always remains cylindrical) or the mass of the polymer in the domain could only be calculated globally and not locally [24]. In the research carried out by Lamberti et al., an attempt was made to overcome the shortcomings of the previous models, such as the affine deformation [25]. Although this model had a high ability to describe the behavior of drug release from hydrogel-based tablets, like the model of Siepmann et al., the mass of the polymer could be estimated globally in the entire matrix domain. It causes the mass value of the components in the model to be unrealistic at some points of the domain [24]. In the research carried out by Caccavo et al. an attempt was made to eliminate the weaknesses of the previous models, which occurred in some of the most important models. Briefly, in the model of Caccavo et al., the equations were considered similar to the model of Lamberti et al., with two main differences: (a) swelling is based on the polymer flux and (b) the mass of the polymer at any point can be estimated locally [24]. Since the simulations in this research are based on the model of Caccavo et al., only a brief explanation of this model is provided and its details are stated in the next section. 

When the phenomenon of drug release is simulated using a powerful mathematical model, it is possible to investigate the effect of different factors on this phenomenon. Usually, a single-point comparison is used to compare two or more drug release profiles (resulting from experimental results or mathematical modeling). For example, the percentages of drug released from several drug carriers are compared at certain time points. The U.S. Food and Drug Administration (FDA) recommends that the use of values that can more comprehensively compare the drug release profiles is preferred [26]. These values can be used to prove the similarity or difference between two drugs with the same purpose. For example, in the research conducted by Kadry et al., they obtained the Dissolution Efficiency (DE) values for the tablets they printed themselves and the tablets available in the market, and using ANOVA tests, they proved that there is no significant difference in DE values among these drugs. In this way, they concluded the bioequivalence of printed drugs with standard drugs available in the market [27]. In addition to the DE value, other comparative measures related to release profile have also been used in past studies. For instance, Hossein et al., to reach the optimal formulation for the release of indapamide, used the amount of Mean Dissolution Time (MDT) [28] and Wei et al. used the values of Area Under the dissolution Curve (AUC) and MDT to evaluate the effectiveness of betaxolol hydrochloride intraocular gel [29].

Most of the studies conducted in this field are related to the effect of drug formulation [13,30,31,32] or the effect of appearance properties or characteristics related to drug production, such as tablet coating weight, porosity, dimensions of particles, the pressure of the tablet compression machine, or many other properties [31,32,33]. One of the factors that can affect the release rate is the geometry of the matrix. However, much fewer studies have studied the effect of matrix geometry on drug release rate compared to the factors mentioned above. In a study conducted by Windolf et al., to investigate the effect of different shapes made by 3D printing technology on the drug release rate, they produced tablets with six different geometries. They by obtained MDT values from the release profiles and, by comparing the surface-to-volume ratio for these shapes and establishing a relationship with the MDT value, concluded that the surface-to-volume ratio has an effect on the drug release rate [34]. In other studies, Karasulu et al. investigated the effect of triangular, hemispherical, and cylindrical geometries on the release profile of theophylline [35], Triboandas et al. investigated the effect of round and oblong geometries produced by hot-melt extrusion technology on the release profile of itraconazole [36], and Molavi et al. also investigated the effect of size and flat face, round convex, and oblong convex geometries on the release profile of domperidone [37].

Therefore, it is stated that although the influence of geometry on the release process has been mentioned in very few articles and in a limited way, it can be further investigated as an effective variable. In most of these studies, the surface-to-volume ratio and, in general, surface-related variables have a significant effect on the drug release rate from tablets. In past studies, the importance of the effect of geometry on the release profile was rarely mentioned and it has never been investigated as an effective factor in determining the responses related to drug release alone. Like the change in the formulation, the ratio of compounds or variables related to tablet production, its geometry, and dimensions have not been looked at as variables capable of predicting the desired responses from the drug release process. Also, based on our knowledge, very few studies have compared common profiles in tablet manufacturing based on release responses. In this research, an attempt is made to fill some of these research gaps.

## 2. Materials and Methods

In general, in this study, the work process is according to the following six steps:

1. One of the mathematical models that can describe drug release behavior from hydrogel-based matrices well was selected for numerical simulations and implemented in a finite element framework.
2. To ensure the model’s and numerical simulation’s validity, the results obtained from the simulations were verified with the experimental results.
3. Several geometries were selected from among the common geometries for oral tablets. The relationship between dimensions and volume was obtained for each geometry, and by fixing the volume, a number of random sizes were obtained for each dimension.
4. Numerical simulation was carried out for each geometry using randomly obtained dimensions, and the percentage of drug released from the matrix until the moment when almost the entire mass of the drug was released was obtained in one-minute time intervals.
5. Using the previous step’s results, the values that show the characteristics of the drug release profile were calculated.
6. These results were interpreted using statistical techniques.

### 2.1. Modeling the Drug Release Process from Hydrogel-Based Tablets

As mentioned in the previous section, the numerical simulations performed in this study are based on the model presented by Caccavo et al. [24,38]. In the following, the details and governing equations of this model will be discussed.

**Figure 1:**
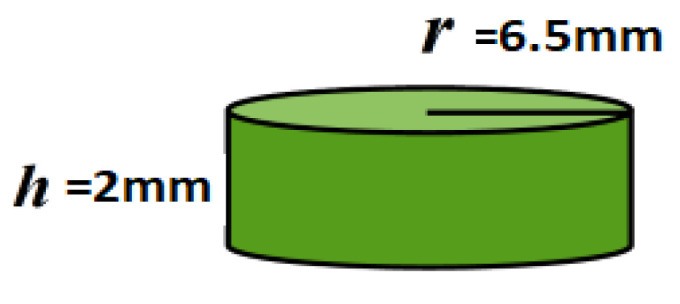
Cylindrical geometry used in the model.

The geometry used in the model is a cylinder with a radius of 6.5 mm and a height of 2 mm (Figure 1).

**Figure 2:**
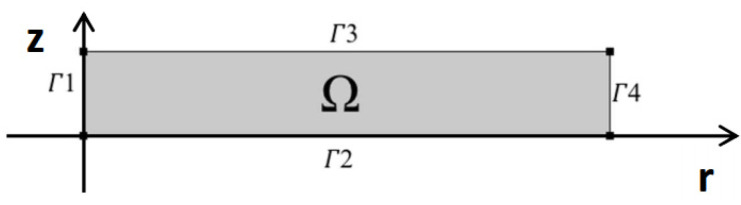
Cross-section and boundaries used in the model [24].

Since the cylinder has an axially symmetric geometry and due to the symmetry condition between the upper and lower half of the cylinder, the simulation was carried out for a rectangle with a radius of 6.5 mm and a height of 1 mm and the symmetry condition was also applied on boundaries 1 and 2 according to Figure 2.

The model generally divides all components in the dissolution process into three components: 1—water, 2—drug, and 3—polymer. Assuming the absence of chemical reaction between particles and regardless of the existence of convection phenomena during the dissolution process, the continuity equation results in the following relationship, which is similar to Fick’s law of diffusion [39].

(1) $$\frac{\partial \rho_{i}}{\partial t}=-\nabla \cdot J_{i}$$

In this relationship, *ρ_i_* and $J_{i}$ are the concentration and flux of the *i*-th component, respectively, and *t* represents time. The important point is that in this modeling, instead of using the concentration variable directly, a transformation according to the following relationship is used:

(2) $$c_{i}=\rho \omega_{i}$$

In this relationship, the concentration of the *i*-th component (*c_i_*) is converted into a coefficient of the average density (*ρ*) and the mass fraction of the i-th component (*ω_i_*) using the above transformation. The average density can also be calculated from the following equation:

(3) $$\frac{1}{\rho }=\frac{\omega_{1}\;}{\rho_{1}}+\frac{\omega_{2}\;}{\rho_{2}}+\frac{\omega_{3}}{\rho_{3}}\;$$

where *ρ*_1_, *ρ*_2_, and *ρ*_3_ are the densities of water, drug, and polymer, respectively. With this transformation, the flux can also be written according to the following relationship:

(4) $$J_{i}=-(\rho D_{i}\nabla \omega_{i})$$

Therefore, the complete relation of diffusion in this model can be rewritten according to the following relationship. This is actually the same as Fick’s law of diffusion but in the form of mass fractions and average density:

(5) $$\rho \frac{\partial \omega_{i}}{\partial t}=\nabla \cdot (\rho D_{i}\nabla \omega_{i})$$

In this relationship, according to what has been stated so far, *ω_i_* is the mass fraction of the *i*-th component, ρ is the average density, and *D_i_* is the diffusion coefficient of the *i*-th component. Rewriting equations based on mass fractions makes it possible to write an obvious relationship between mass fractions at any point:

*ω*_3_ = 1 *− ω*_1_
*− ω*_2_ (6)

In this relationship, *ω*_1_, *ω*_2_, and *ω*_3_ are water, drug, and polymer mass fractions, respectively. This self-evident relationship causes the mass fraction and, ultimately, the concentration of the polymer component at any point of the domain to be written in terms of the mass fractions of water and drug, and therefore solving the equations related to water and drug expresses all the necessary information at any point of the domain. Therefore, there is no need to solve equations for the third component, i.e., polymer. To solve these equations, we need initial and boundary conditions. At the initial moment (t = 0), at all points of the domain, the mass fractions of drug and polymer are equal to 0.5 and for water it is equal to 0. This is because, before the contact of water and matrix, there is clearly no water in the matrix and the matrix is completely dry. Also, the proportions of polymer and drug composition in the matrix are equal.

(7) $$t=0\;\;\;\;\;\forall x\;\epsilon \;\Omega :\;\;\omega_{i}=\omega_{i0}$$

In this relation, *ω_i_*_0_ represents the mass fraction of the *i*-th component in the matrix at the starting moment of the dissolution process. This value equals 0.5 for drug and polymer and 0 for water. Regarding the boundary conditions, it is assumed that there is only water on boundaries 3 and 4 (Figure 2), which are in contact with water, where the amount of drug is zero. It means that the mass fraction of water must be one on the outer borders of the matrix and zero for the drug. However, in this model, a 3% share for the polymer on the boundaries is considered, and therefore the mass fraction of water is equal to 0.97, and the mass fraction of the drug is 0:

(8) $$t>0\;\;\;\;\;\forall x\;\epsilon {\;\Gamma }_{3}\;\&{\;\Gamma }_{4}:\;\;\;\;\;\omega_{i}=\omega_{ieq}$$

In this relationship, *ω_ieq_* represents the mass fraction of the *i*-th component while the system is in equilibrium. More clearly, equilibrium means when all the drug inside the matrix is dissolved in water and almost all the remaining small volume of the swollen matrix is also composed of water. Since the symmetry condition is considered on boundaries 1 and 2 (Figure 2), the flux entering these two boundaries is considered zero (Relation (9)). The symmetry condition on boundary 1 is due to axial symmetry and on boundary 2 it is due to the physical symmetry of the cylinder. Thus, only half of it is modeled for this reason.

(9) $$t>0\;\;\;\;\;\;\;\;\forall x\;\epsilon {\;\Gamma }_{1}\;\&{\;\Gamma }_{2}:\;\;\;\;J_{i}=0$$

The last point about solving diffusion equations is related to the diffusion coefficient. If the diffusion coefficient is not constant, it is usually considered concentration dependent. However, it can also depend on time or coordinates. If the diffusion coefficient is not assumed to be constant, it can help make the problem more realistic. In this modeling, the diffusion coefficient depends on the water concentration at any domain point. With this interpretation, the higher the water concentration at a point, the higher the diffusion coefficient; therefore, more, faster diffusion occurs [39]. This assumption is logically acceptable because when there is more water in a space, obviously, particles can diffuse more easily and at a higher speed. In this modeling, the diffusion coefficient is considered as:

(10) $$D_{i}=D_{\mathrm{ieq}}\;[-\beta_{i}(\frac{\omega_{1}}{\omega_{1\mathrm{eq}}})]$$

in which *D*_ieq_ is the diffusion coefficient in the equilibrium state of the system. *β_i_* is also a positive value for the *i*-th component, determined using experimental results through trial and error. According to (10) and the previous explanation, the diffusion coefficient for each component reaches its maximum value, *D*_ieq_, if the mass fraction of water is equal to its maximum value, i.e., ω_1eq_ = 0.97, but if the mass fraction of water takes its minimum value, which is zero (which occurs at the initial moments), the diffusion coefficient is equal to its minimum value, $\frac{D_{\mathrm{ieq}}}{e^{\beta i}}$. 

#### 2.1.1. Modeling Swelling and Erosion

As mentioned before, one of the strengths of this modeling is the ability to describe swelling and erosion phenomena. For this purpose, deformation equations are applied to the domain according to the following relations.

(11) $$\begin{array}{c} \frac{\partial^{2}}{\partial R^{2}}\left(\frac{\partial r}{\partial t}\right)+\frac{\partial^{2}}{\partial Z^{2}}\left(\frac{\partial r}{\partial t}\right)=0\\ \frac{\partial^{2}}{\partial R^{2}}\left(\frac{\partial z}{\partial t}\right)+\frac{\partial^{2}}{\partial Z^{2}}\left(\frac{\partial z}{\partial t}\right)=0\; \end{array}$$

where *R* and *Z* represent the material coordinates in the radial and vertical directions, respectively, and *r* and *z* represent the spatial coordinates in the radial and vertical directions, respectively. The deformation of the domain is calculated based on Equation (11), which is the Laplace smoothing equation. To solve this partial differential equation, the initial condition is defined according to the following equation.

(12) $$t=0\;\;\;\;\;\left(r\cdot z\right)=\left(R\cdot Z\right)=\left(r_{0}\cdot z_{0}\right)$$

This relationship states that at the initial moment (*t* = 0), the spatial and material coordinates coincide. The following boundary conditions are also used to solve these equations:

(13) $$t>0\;\;\;\;\;\forall x\;\epsilon {\;\Gamma }_{3}{\;\mathrm{or}\;\Gamma }_{4}:\;\;\;\;v=\left(v_{swe}+v_{er}\right)$$

In this relationship, v is the speed of the boundary movement, $v_{swe}$ denotes the surface swelling speed, and $v_{er}$ stands for the erosion speed (which will be explained later). For boundaries 1 and 2, according to the symmetry condition, it is assumed that each boundary has no movement in its perpendicular direction according to the following relationship.

(14) $$\begin{array}{c} t>0\;\;\;\;\;\;\forall x\;\epsilon {\;\Gamma }_{1}:\;\;\;\;\;dr=0\\ t>0\;\;\;\;\;\forall x\;\epsilon {\;\Gamma }_{2}:\;\;\;\;\;dz=0\; \end{array}$$

**Figure 3:**
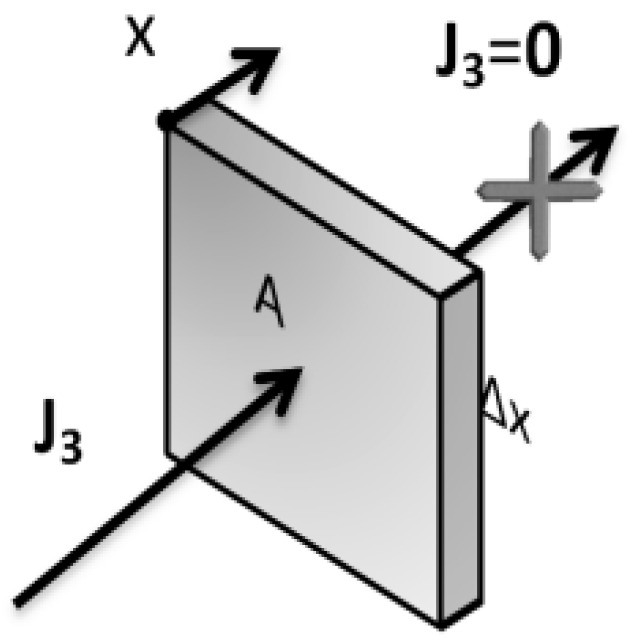
Polymer flux on the surface element at the erosion front [24].

The value of v is the speed of movement of the boundaries, caused by the polymer flux at any boundary point. Caccavo et al. assumed that the speed of movement of the boundaries is directly related to the polymer flux at each point. In this way, it is assumed at every point that the sum of the passing flux of all three components must be equal to zero (Figure 3 and Relation (15)).

(15) $$\sum J_{i}=0\;$$

The polymer flux can also be related to the boundary movement speed as:

(16) $$J_{3}A=\rho \omega_{3}A\frac{dx}{dt}\;$$

The above relationship states that a mass of polymer that passes through a surface of area *A* in one second causes the outer surface of the matrix to move forward. The amount of thickness that the polymer mass creates behind surface *A* in one second is calculated as:

(17) $$\frac{dx}{dt}=\frac{J_{3\;}}{\rho \omega_{3\;}}\;$$

To fully understand the above relationship, it should be remembered that the term *ρ*ω_3_ is the concentration of the polymer. Combining Relations (15) and (17), the value of $v_{swe}$ can be calculated:

(18) $$v_{swe}=\frac{dx}{dt}=\frac{J_{3\;}}{\rho \;\omega_{3}}=\frac{-\left(J_{1}+J_{2}\right)}{\rho \;\omega_{3}}\;$$

In order to also account for the erosion phenomenon, it is assumed that due to the interaction of the solvent and the polymer on the surface of the matrix, the polymer dissolves in water at a low speed on the contact surface with water and also causes a decrease in the volume of the matrix. It is also assumed that this effect of the solvent on the polymer will always be constant on the surface of the matrix. This speed is applied as a negative value (the direction is inside the domain) on surfaces 3 and 4 (Figure 2) according to the following relationship:

(19) $$v_{er}\cdot n=-K_{er}$$

In this relationship, $K_{er}$ is the erosion constant and $v_{er}$ is the erosion speed. This erosion constant means the rate at which the outer boundaries of the matrix are continuously eroding due to contact with the dissolution medium.

Using the sum of two velocities for swelling and erosion at the same time makes the simulation results closer to the experimental results. In practice, it can be seen that as soon as the dissolution process begins, the matrix volume increases, but this rate of increase gradually decreases, and until the complete dissolution of the matrix, the volume change will have a decreasing trend. According to Relation (18), it is also deduced that during this time, the concentration gradient of drug and water between the external environment and the inside of the matrix decreases, and as a result, the rate of volume increase decreases. When the concentration gradient reaches zero, according to this relation, there should be no change in shape, while in practice we will have a decrease in volume at the end of the dissolution process. Therefore, it becomes necessary to use a velocity in the inner direction of the matrix ($K_{er}$) that can describe the volume reduction. In addition, due to the contact between the surface of the matrix and the water environment, it is expected that during the dissolution process a part of the polymer on the surface will constantly dissolve in water. To estimate an initial value for the erosion constant, one can use the experimental reduction rate of the polymer mass in the tablet over time [25].

#### 2.1.2. Model Parameters

Solving the equations in the model requires a series of input values, some of which are determined depending on the material used and some of which must be determined by trial and error. The water and drug mass fractions at the initial moment (ω_10_ and ω_20_), water and drug mass fractions in the equilibrium state (ω_1eq_ and ω_2eq_), and the water, drug, and polymer densities (*ρ*_1_, *ρ*_2_, and *ρ*_3_) are values which should be entered into the modeling according to the materials used, the ratio of compositions, and boundary conditions. *D*_1*eq*_, *D*_2*eq*_, β_1_, β_2_, and *K_er_* are five values that need to be calibrated by experimental results. Although *D*_1*eq*_ and *D*_2*eq*_ also have physical interpretations, Caccavo et al. point out that these values should be calibrated anyway, and the experimental results can only provide values to start the process of trial and error [24]. Therefore, in order to use this model to simulate the behavior of a hydrogel-based drug delivery system, it is necessary to fit these values by a series of results obtained from experimental tests. The values used in the simulations in this study are presented in Table A3. It should be noted that all the simulations in this study were carried out using the values related to the speed of 75 rpm in Table A3.

### 2.2. Validation of the Model

**Figure 4:**
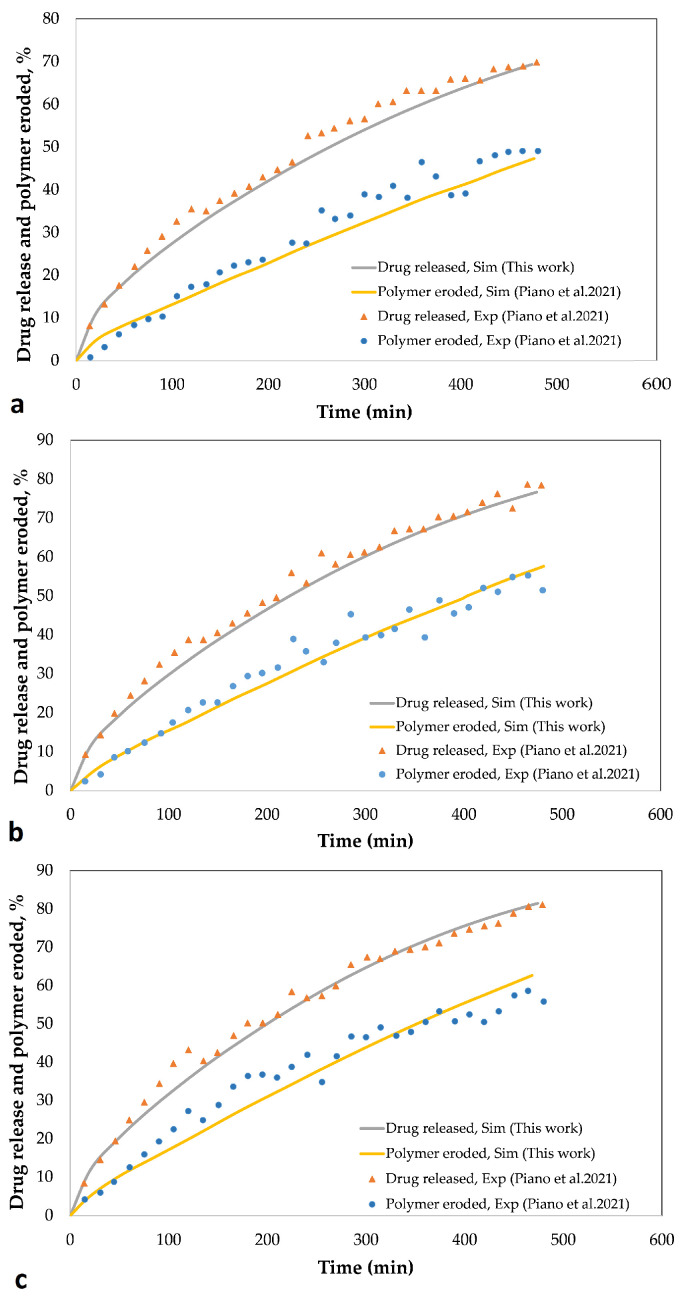
Percentage of the drug released and the polymer eroded during the time while rotating the paddle at a speed of (**a**) 50 rpm, (**b**) 75 rpm, (**c**) 100 rpm. Drug released, Sim: the percentage of drug released obtained from the simulation, Drug released, Exp: the percentage of drug released obtained from the experimental results, Polymer eroded, Sim: the percentage of polymer eroded obtained from the simulation, Polymer eroded, Exp: the percentage of polymer eroded obtained from the experimental results [40].

In order to be able to confidently interpret the results of the simulations, one must first ensure that the simulation results are close to the experimental ones. It is not easy to find experimental results that can provide all the basic information needed for simulation, because very few articles describe all the details of the polymer and drug composition ratio and their type, matrix dimensions, and density of all materials used. For this reason, a handful of experimental results can be used as a reference for validating the simulation. In this research, the experimental results presented by De Piano et al. published in 2021 [40] and the experimental results presented in the research by Lamberti et al. [25] have been used. The first study [40] presents the experimental results for three rotation speeds of the paddle in the dissolution test process. These results include the percentage of drug release and the percentage of polymer eroded over time (Figure 4). It can be seen that in all the graphs (Figure 4), including the drug release and polymer erosion, the results of the simulations performed in this study are very close to those of experiments, which indicates the acceptability of the simulation results. In Figure 4, it can be seen that with the increase in the rotation speed of the paddle, with the passage of time, the percentage of released drug and eroded polymer increases slightly. To simulate the dissolution process with different rotation speeds of the paddle, it is enough to increase the erosion constant by increasing the rotation speed of the paddle and the rest of the model parameters remain intact (Table A3).

**Figure 5:**
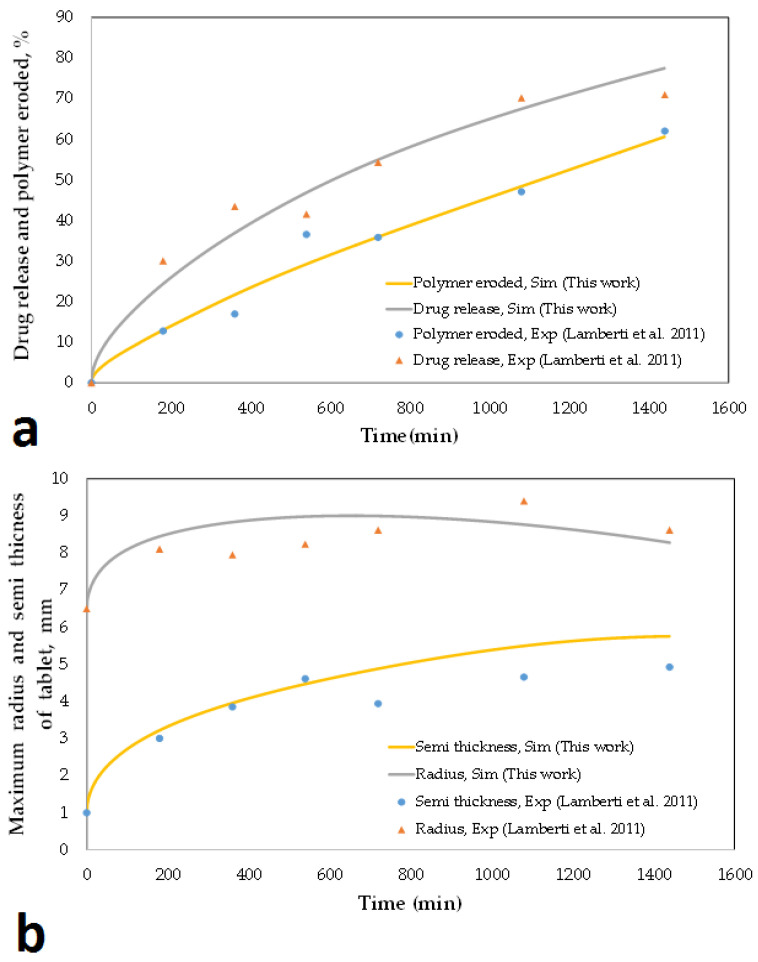
(**a**) Percentage of the drug released and polymer eroded during the time and (**b**) maximum radius and half-thickness of tablet during the time. Drug release, Exp: the percentage of drug released obtained from the experimental results, Drug release, Sim: the percentage of drug released obtained from the simulation, Polymer eroded, Sim: the percentage of polymer eroded obtained from the experimental results, Polymer eroded, Exp: the percentage of polymer eroded obtained from the simulation, Semi thickness, Sim: semi-thickness of tablet obtained from the simulation, Semi thickness, Exp: semi-thickness of tablet obtained from the experimental results, Radius, Sim: radius of tablet obtained from the simulation, Radius, Exp: radius of tablet obtained from the experimental results [25].

Since in this article, like most studies, the results of shape change have not been discussed, in the following, employing the information provided in the research carried out by Lamberti et al. [25], in addition to the drug release and the percentage of eroded polymer, it is attempted to fit the deformation of the matrix. In Figure 5, it can be seen that in addition to the percentage of drug release and polymer erosion, the values of the maximum height and maximum radius of the matrix are also in good agreement with the simulation results. Therefore, using the set of results presented from these two articles, it is shown that the simulations have a good compatibility with the experimental results.

### 2.3. Geometries

As mentioned earlier, the aim of this study is to examine the effect of tablet geometry on the drug release process. For this purpose, eight different and common geometries in the production of oral tablets were investigated. All the selected geometries are round tablets, so these geometries have axial symmetry, like the geometries that were examined in the research of Caccavo et al. and other researchers [24,38,40]. In this study, to make a correct comparison, the mass of the tablets should be kept constant. According to past studies that showed that tablets’ size affects the release profile [37] and Caccavo et al. [38], who made a comparison between several release profiles, all tablets had the same mass. Since the ingredients and composition ratio are assumed to be the same for all the tablets, they have the same density. By assuming constant density, it is necessary to consider a constant volume to keep the mass of tablets the same. Since all investigated geometries have axial symmetry, using Pappus’s theorem, the general relationship of volume can be determined based on the dimensions of each geometry (Table 1). The eight examined geometries are: 1—shallow convex, 2—standard convex, 3—deep convex, 4—extra-deep convex, 5—flat face, 6—flat face beveled edge, 7—ring or torus, and 8—core rod. In the following, these geometries will be written as 1—SHC, 2—STC, 3—DC, 4—EXDC, 5—FF, 6—FFBE, 7—TO and 8—CR, respectively, for briefness. Table 1 gives the volume relationships based on the dimensions of each geometry’s cross-section.

## 3. Results

**Figure 6:**
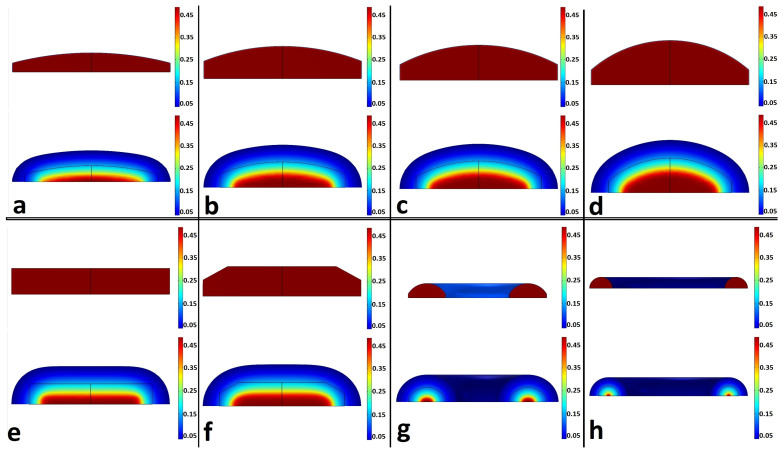
Drug mass fraction and matrix shape change in (**a**) SHC, (**b**) STC, (**c**) DC, (**d**) EXDC, (**e**) FF, (**f**) FFBE, (**g**) CR, (**h**) TO geometries, before the start of the dissolution process (**upper** figures) and 6 h from the start of the dissolution process (**lower** figures).

After the drug release process was simulated for the desired geometries (Figure 6) and the release profile was obtained for each one, the results were categorized into three parts: 1—results related to each geometry, 2—results related to common features, and 3—comparing geometries with each other. In the first part, the aim is to answer whether, knowing the dimensions of a geometry and its type, it is possible to predict the expected responses of a release profile with appropriate accuracy. For each type of geometry, the drug release process for 20–40 matrices with different dimensions was simulated, and statistical modeling was used to check how accurately the responses of a release profile can be predicted by having the dimensions of a matrix.

The second part relates to whether the common features of the matrices, such as the maximum length, maximum width, or surface-to-volume ratio, have a significant effect on the release profile. This question is also answered by applying statistical modeling, showing that regardless of the type of geometry and simply by having these values in hand, it is possible to make an accurate prediction of the responses resulting from the drug release profile. The reason for presenting this part is that in addition to the fact that the effect of these values on the release profile has been emphasized in past studies [34,37,38], these common features are of special importance in drug design for purposes such as patient compliance [41]. Thus, this issue is important from the point of view of designing tablets. In the third part, the question whether there is a significant difference between the types of geometries in the responses caused by the release profiles or not is answered. It is noteworthy that all these simulations were carried out by keeping the mass and dose of the drug fixed. This means that if the results of this part are meaningful, during the design of a drug tablet, by only changing the dimensions or the type of geometry and without any change in the dose and the polymer carrier type, according to the drug class and the needs of the patients, the release profile can be adjusted to the target profile as much as possible. It should also be noted that the following parameters are extracted from the release profile: a. 95 rel (the time required to release 95% of the initial mass of the drug), b. 30 min (percentage of drug released after 30 min from the start of the dissolution process), c. area under the dissolution curve (AUC), d. dissolution efficiency (DE), e. mean dissolution time (MDT), and f. variance of dissolution time (VDT). *DE*, *MDT*, and *VDT* are obtained as [42]:

(20) $$DE=100\times \frac{\int_{0}^{t}y\;dt}{y_{100}\;t}\;$$

(21) $$\;MDT=\frac{\sum_{i=1}^{i=n}\;\bar{t\;}\Delta M_{i}}{\sum_{i=1}^{i=n}\Delta M_{i}}$$

(22) $$VDT=\frac{\sum_{i=1}^{i=n}\left(\bar{t\;}-MDT\right){\;}^{2}\;\left(\Delta M_{i}\right)}{\sum_{i=1}^{i=n}\Delta M_{i}}\;$$

In these relations, y is the amount of drug released until time t, $y_{100}$ is the amount of drug released at the last time of recording the release result, Δ*M_i_* means the percentage of drug released between times t_i_ and t_i+1_, and $\bar{t}$ is the average of t_i_ and t_i+1_.

### 3.1. Results Related to Each Geometry

Since the tablets’ mass and volume have been kept constant in all the simulations, this causes a strong dependence between the dimensions in all the investigated geometries. If there is a strong dependence between the independent variables in a linear regression model, the results are greatly weakened, and if this dependence exceeds a certain value, the results lose their validity. One of the regression methods that can be used in these cases is partial least squares (PLS) regression [43]. To estimate the power of the represented models, we use the values of coefficient of determination (R^2^) and the mean absolute percentage error (MAPE) simultaneously. In Table 2, the values of R^2^ and MAPE for all the built models that include 48 regression equations, as well as the lower and upper limits of the ranges, are presented. It is revealed that, for example, by having the exact dimensions of the FFBE type geometry, on average, the six examined responses can be predicted with a coefficient of determination of 0.978, while the deviation from the values obtained from the simulations is only about 0.66%. For other geometries, the predictions are approximately as strong as mentioned. It is also inferred that, in general, by having the dimensions of a geometry, with an R^2^ of 0.96 on average, the responses related to release can be accurately predicted, while there is about 1.27% deviation from the responses. Among the responses, the lowest average prediction accuracy is for DE, which has an accuracy of about 0.92, while the other responses have coefficients of determination close to 1.

### 3.2. Results Related to Common Features

During the design of a drug tablet, special attention is paid to features such as maximum length, apparent surface area, or apparent volume because these values can be directly related to patient compliance [41]. Since these features can be checked for any tablet and with any geometry, it is possible to check all eight chosen geometries simultaneously, this time using common dimensional features. Another issue is the special importance of surface-related variables in the diffusion process. Many studies have emphasized the importance of variables such as surface or surface-to-volume ratio in the release process [35,44]. Therefore, in addition to the mentioned features, the effect of variables related to surface area is also investigated in this section, because in some of the previous research, the reason for the difference in drug release profiles between different forms of tablets has been justified by these variables [37].

According to the past studies and, of course, the introduction of the variable “ratio of maximum height to maximum length”, the following four variables were examined:

1. The ratio of surface area to volume (S/V) [44];
2. The total length of dimensions (LWH) [41];
3. Apparent volume (APV) [41];
4. Ratio of maximum height to maximum length (H/L).

**Figure 7:**
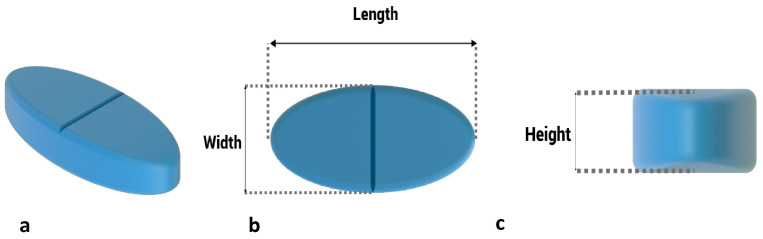
Common features of tablets. (**a**) Perspective, (**b**) maximum length and maximum width, (**c**) maximum height.

In the case of the first variable, the ratio of surface area to volume, it is enough to divide the surface area by volume. Variable number 2 is calculated as shown in Figure 7. Of course, since all the geometries examined in this research are round tablets, their length and width are the same due to axial symmetry. Variable number 3 is the product of maximum length, width, and height, as shown in Figure 7. The apparent volume is always greater than the real volume, and only if the geometry is a cube are these two values equal.

In addition to the previous three variables that have been considered in previous studies, the variable of the ratio of maximum height to maximum length has also been investigated. To calculate this parameter, it is enough to divide the height by the length according to Figure 7, and as a result, a dimensionless variable is obtained. Correlations between variables and responses are specified in Table A1. It is observed that all the correlations are significant at the significance level of 0.01. Regarding the signs, it is also observed that, for example, as expected, the higher the surface-to-volume ratio, the shorter the release time of the entire drug mass because the sign of the correlation between the two is negative. It can be seen that, on average, the surface-to-volume ratio is the most effective variable, with an average correlation value of 0.89. According to Table A1, it is concluded that all considered variables have a significant effect on the estimation of responses related to the release. Now, the second question must be answered: Is it possible to accurately predict the drug release profile by having these values, as in the previous part?

In this section, due to the strong correlation between variables, partial least squares regression is used so that we can ignore multicollinearity. Table 3 provides the details of statistical modeling for all six responses. According to the values of the coefficient of determination and the mean absolute percentage error in Table 3, the most suitable prediction is related to the response of “30 min”. This variable is presented as a representative of burst release. This high predictive power shows that by having common features, regardless of the type of geometry, the amount of burst release can be predicted, and the type of geometry cannot significantly affect it. But according to other responses, it can be seen that the coefficient of determination is about 0.90 on average, while on average, there is about 13.9% error in the prediction values. These values are significantly less accurate than the previous part of the results, i.e., modeling based on geometry dimensions, because in the previous part, the same values were about 0.96 and 1.5%, respectively, while in many studies, variables related to the surface were introduced as the determining factor in estimating the responses related to the diffusion process. In this part, in addition to this variable, three other variables were also added for modeling, but even so, the models could not provide significant accuracy. The important result is that the surface factor alone cannot accurately predict the release process and the type of geometry significantly affects these responses. Now another question arises as to why, contrary to what was expected, these characteristics, especially the surface factor, cannot be good regressors in the diffusion process. The following results can clarify this issue.

**Figure 8:**
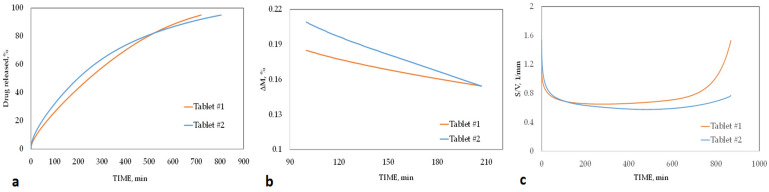
(**a**) Cumulative percentage of the drug released, (**b**) percentage of the drug released at each moment, (**c**) surface-to-volume ratio during the time for tablet #1 (orange line) and tablet #2 (blue line).

The two series of given information correspond to two matrices of two different geometries (Table 4). In general, it is expected that the higher the ratio of the surface to the volume of a matrix, the less time it will take for the entire mass of the drug to be transferred to the dissolution medium, because more surface area results in faster release. It can be seen that the surface area (S) and the surface-to-volume ratio (S/V) for matrix #1 are smaller than the same values for matrix #2, but the release of the entire drug mass (95 rel) for matrix #2, contrary to expectation, requires more time. But the amount of burst release (30 min), as expected, is higher for the matrix with more surface area, i.e., matrix #2. Therefore, some responses are in line with our expectations and some are against our expectations. By looking at the variance of dissolution time (VDT) values, it is clear that the drug release process for matrix #2 is a far from linear process, and the percentage of drug released at the time intervals is very different. Therefore, the release rate frequently changes. In order to have a better understanding of the drug release process in these two matrices, we obtain help from Figure 8a–c. It can be seen in Figure 8a that initially the amount of drug released from matrix #2 is more than that of matrix #1. Therefore, as expected, the percentage of drug released in the first 30 min is higher for matrix #2, which has a higher surface-to-volume ratio. However, between 500 and 600 min, the two lines cross each other, and from then on, the percentage of drug released from matrix #1 is higher than matrix #2. Now it is better to examine the release rate. In Figure 8b we see the percentage of the drug released at each moment. It is observed that in the initial moments, the rate of drug release from matrix #2 is higher, but gradually and a little after 200 min, the rate of drug release from matrix #1 surpasses it. Therefore, we can now state that the reason for the longer duration of complete release of the drug from matrix #2 is that after the first few minutes, the release of the drug from matrix #1 gradually speeds up, and the cumulative amount of drug released after about 520 min is more than the amount of drug released from matrix #2. But this increase in the release rate itself is not without reason. Figure 8c shows the changes in surface-to-volume ratio against time. In this graph, it can be seen that in the initial moments, the ratio of surface to volume in matrix #2 is more than this value in matrix #1, but almost 100 min from the start of dissolution, the two curves cross each other, and gradually it is matrix #1 which has a higher surface-to-volume ratio. It was also pointed out in previous studies that the change in the shape of the matrix over time has a significant effect on the drug release process and can produce unexpected results [38,45]. Therefore, these results show from another point of view that the difference in geometry can have a direct and significant effect on the drug release process. This means that it is difficult to predict how the shape of each geometry will change at different moments, and they cannot be identified from the primary features. This section states how our prediction of a drug release profile can be wrong and far from reality without having the type of geometry and only relying on variables such as surface.

### 3.3. Comparison of Geometries

In this part, we try to compare the geometries based on their responses from a general point of view. To describe the difference in the average responses in different geometries, we must first measure the significance of these differences. For this, we use one-way ANOVA tests to interpret their results from several perspectives. In Table A2, the *p*-values (sig) indicate that the difference in means for all six investigated responses is significant. But if the average responses have a significant difference only between two groups of the eight investigated geometries, the result of the ANOVA test will show significance. To find out which differences between these groups are significant, we must use post hoc tests. The Tamhane post hoc test is used in this research. To have a better view of the results, the results of this test are completely summarized in Table 5. The sign “✓” means the difference is significant and the sign “-” means the difference between the averages of two groups is not significant. All the signs on the diagonal line of Table 5 indicate the lack of significance of the difference because, clearly, the difference in values between two identical groups is always meaningless. Therefore, a total of 28 comparisons have been made for each of the responses (168 comparisons in total). It should be noted that the level of significance in all investigated cases is set at 0.05. Comparing these results, one finds that all responses, except DE, have significant differences in 22 to 24 out of 28 possible situations. It means that only by changing the type of geometry and with the same amount of drug and without the slightest change in the formulation, the time required to release the entire mass of the drug (95 rel), the time the drug remains in the bloodstream (AUC), the ability of the matrix to keep the drug in dosage form (MDT), and the percentage of the burst release of the drug (30 min) change significantly. But the conditions for the DE response are distinct and significant differences were obtained in only 10 cases. Therefore, as stated in the first part of the results, this response is not particularly sensitive to geometry change. It also means that, contrary to the responses mentioned, in most cases, by changing the type of geometry, the bioavailability of the drug does not undergo significant changes. In general, except for DE, the other five responses undergo significant changes by changing the type of geometry of the matrix.

**Figure 9:**
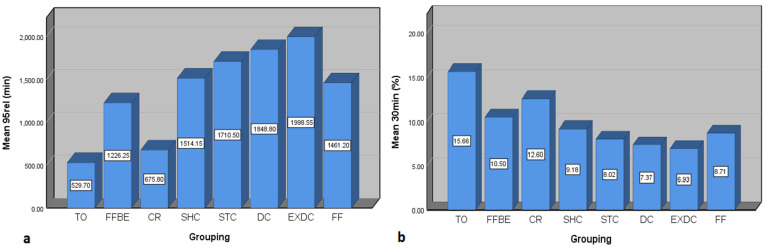
Comparison of average (**a**) 95 rel response (the time required to release 95% of the initial mass of the drug) and (**b**) 30 min response (percentage of drug released after 30 min from the starting moment of the dissolution process) for the 8 investigated geometries.

In the case of the 95 rel response, the range of changes was 374 to 2267 min (Table 2). This means that in some cases, the time required to release the total drug between two geometries is different by about six times. It should be remembered that this difference occurs between two matrices with the exact same formulation and dosage. Thus, once again, these results show that the change in tablet geometry significantly affects the release-related responses. The results show that, on average, EXDC and TO geometries require the maximum and minimum time to release all the initial mass of the drug, respectively. More completely, the order of geometries with the maximum to minimum time required to release all the initial mass of the drug is as follows: 1—EXDC, 2—DC, 3—STC, 4—SHC, 5—FF, 6—FFBE, 7—CR, and 8—TO (Figure 9a). Regarding the 30 min response, the range of changes was from about 6.5 to 19.7 percent (Table 2). This amount of difference between the upper and lower limits of the interval is also remarkable considering the how little time is considered after the dissolution start moment, especially considering that, in general, the response related to burst release is of special importance from a biological point of view. The results show that, on average, TO and EXDC geometries have the maximum and minimum percentage of burst release, respectively. More completely, the order of geometries with the maximum to minimum percentage of burst release is as follows: 1—TO, 2—CR, 3—FFBE, 4—SHC, 5—STC, 6—FF, 7—DC, and 8—EXDC (Figure 9b). The order of the average response for VDT can also be important because the lower the VDT value, the closer the release profile is to the zero-order release curve, and the higher the value, the further away it is from this curve. On average, among the geometries, EXDC and TO have the highest and lowest amount of VDT, respectively. Therefore, the TO geometry has the closest behavior to the zero-order release behavior.

The order of the average of the six investigated responses for the groups (eight geometries) is almost the same as the results that were expected according to the average values of surface-to-volume ratios. But, in some cases, it is different. For example, the average surface-to-volume ratio for SHC and FF geometries is 1.0 1/mm and 0.97 1/mm, respectively. It seems that the higher this value is, the time required to release 95% of the initial drug should be less, while the average time required to release 95% of the initial drug for SHC and FF geometries is 1514 and 1461 min, respectively (Figure 9a). Similarly, in some cases, expectations about other responses are violated by comparing geometries. Therefore, once again, the results confirm that, despite the significant effect of surface-related variables on the drug release process, the final results do not always match the expectations caused by these variables. Therefore, not only on a case-by-case basis but also with a general look at the types of geometries, the difference in the geometry type produces unexpected results in drug release profile responses. 

## 4. Conclusions

In this research, an attempt was made to investigate the effect of tablet geometry on the release profile with a numerical approach. For this purpose, the release process of two hundred tablet samples, which included eight commonly used geometries in the production of oral tablets, was simulated, and the release profiles were obtained. The results showed that, on average, these responses can be predicted with a determination coefficient of about 0.96 and a percentage error of about 1.3%. It means that the drug release profile can be predicted with high accuracy only by knowing the type of geometry and its dimensions without conducting numerous dissolution tests. Although such a result has been obtained for variables such as formulation [13,30,32] or appearance properties and variables related to drug production [31,32,33] with an experimental approach in previous studies, based on our knowledge, these results for different types of geometries and their dimensions have not been mentioned in previous studies. Another result was that while having common features of tablets such as surface-to-volume ratio or maximum tablet length, regardless of the type of geometry and despite the significant effect of all these common features on release profile responses, it is not possible to have a powerful prediction of the drug release profile. This is due to the fact that the difference in geometries causes unpredictable results, and the shape change in the tablets during the dissolution process can proceed in such a way that a tablet with a higher surface-to-volume ratio at the beginning of the dissolution process will gradually lose this characteristic compared to tablets that initially had a smaller surface area. This result was implicitly stated in some studies [38]. By comparing the eight tested geometries based on the responses caused by the release profile, such as the ability to keep the drug in the dosage form, the time the drug remains in the bloodstream, the time required to release the entire mass of the drug, and the amount of burst release, we conclude that all the responses, except the dissolution efficiency (DE), are sensitive to the change in the type of geometry without any changes in dose or the mass of the drug.

## Figures and Tables

**Table 1:** Volume relationships in terms of dimensions for geometries.

Geometry	Cross Section	3D view	Volume in Term of Dimensions
SHC, STC, DC, EXDC	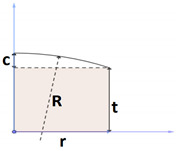	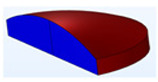	$\pi \left(R^{2}c+\frac{{\left(R-c\right)}^{3}}{3}+r^{2}t-\frac{1}{3}R^{3}\right)$
FF	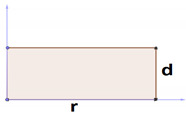	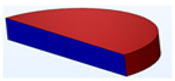	$\pi r^{2}t$
FFBE	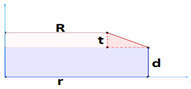	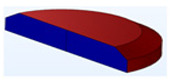	$\pi \left(r^{2}d+\frac{t{\left(r^{2}+rR-2R^{2}\right)}^{3}}{3}+R^{2}t\right)$
TO	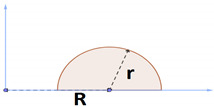	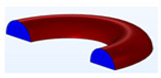	$\pi r^{2}R$
CR	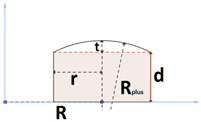	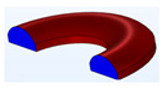	$\pi R\left({R_{plus}}^{2}\left(Arc\sin\left(\frac{2r(R_{plus}-t)}{\;{R_{plus}}^{2}}\right)-\frac{2r\left(R_{plus}-t\right)}{{R_{plus}}^{2}}\right)+4rd\right)$

**Table 2:** Values of coefficients of determination (R^2^), mean absolute percentage error (MAPE), and intervals for 48 constructed regression equations.

Geometry		95 rel	30 min	AUC	DE	MDT	VDT	Mean
SHC	R^2^	0.860	0.870	0.860	0.770	0.870	0.840	0.845
	MAPE	5.670	6.130	5.350	0.600	6.470	14.270	6.415
	lower bound	1049.000	7.035	70,128.714	58.873	308.782	74,225.856	
	upper bound	2151	13.41291	136,269.14	62.39242	714.4524	331,213.4941	
STC	R^2^	0.990	0.990	0.990	0.980	0.990	0.990	0.988
	MAPE	0.420	0.550	0.360	0.090	0.570	1.050	0.507
	lower bound	1257.000	6.951	82,373.604	58.818	387.781	107,238.422	
	upper bound	2117	10.88369	133,893.438	60.63912	705.4269	319,899.3878	
DC	R^2^	0.990	0.990	0.990	0.940	0.990	0.990	0.982
	MAPE	0.180	0.430	0.170	0.070	0.420	0.410	0.280
	lower bound	1482.000	6.754	95,185.001	58.600	477.900	151,145.561	
	upper bound	2196	8.806506	138,737.114	59.39904	733.4534	345,891.8416	
EXDC	R^2^	0.980	0.900	0.980	0.900	0.980	0.990	0.955
	MAPE	0.790	1.370	0.780	1.320	0.910	1.700	1.145
	lower bound	1682.000	6.523	106,443.537	58.662	559.356	198,202.001	
	upper bound	2267	7.620341	141,811.315	59.58809	772.0898	373,557.7719	
CR	R^2^	0.990	0.950	0.990	0.870	0.990	0.990	0.963
	MAPE	0.490	1.200	0.460	0.180	0.650	1.350	0.722
	lower bound	586.000	11.094	35,658.428	58.530	208.521	25,995.125	
	upper bound	781	14.04352	47,111.6423	59.89977	283.4317	46,229.25358	
FF	R^2^	0.990	0.990	0.990	0.980	0.990	0.990	0.988
	MAPE	0.019	0.094	0.026	0.013	0.200	0.024	0.063
	lower bound	1301.000	8.012	83,685.395	59.130	418.040	114,779.506	
	upper bound	1627	9.575512	103,369.908	59.36075	536.825	184,766.2872	
FFBE	R^2^	0.990	0.980	0.990	0.930	0.990	0.990	0.978
	MAPE	0.350	1.890	0.260	0.360	0.600	0.500	0.660
	lower bound	805.000	7.312	53,521.067	58.712	239.565	44,790.424	
	upper bound	1759	15.24185	111,233.695	63.14603	586.0399	217,187.2251	
TO	R^2^	0.990	0.990	0.990	0.990	0.990	0.990	0.990
	MAPE	0.140	0.200	0.130	0.080	0.190	1.490	0.372
	lower bound	374.000	10.868	23,061.813	58.568	129.222	10,500.601	
	upper bound	809	19.696	48,917.2535	64.71681	292.0741	49,315.18992	
Mean	R^2^	0.973	0.958	0.973	0.920	0.974	0.971	0.961
	MAPE	1.007	1.483	0.942	0.339	1.251	2.599	1.270
Lower bound		374.000	6.523	23,061.813	58.530	129.222	10,500.601	
Upperbound		2267	19.696	141,811.315	64.71681	772.0898	373,557.7719	
Upperbound/Lowerbound		6.061497	3.019251	6.14918319	1.105705	5.97491	35.57489361	9.6476

**Table 3:** R2 and MAPE of the regression models built for 6 responses based on the variables of common features.

	95 rel (min)	30 min (%)	AUC (min)	DE (%)	MDT (min)	VDT (min^2^)	Mean
R^2^	0.880	0.980	0.860	0.800	0.920	0.950	0.898
MAPE	17.440	2.370	19.610	0.650	13.630	29.800	13.917

**Table 4:** Surface amount, surface-to-volume ratio, and release profile responses for tablets #1 and #2.

	S (mm^2^)	S/V (1/mm)	95 rel (min)	30min (%)	AUC (min)	DE (min)	MDT (min)	VDT (min^2^)
Tablet #1	153.7856397	1.158615141	720	11.82085725	43,453.9542	58.78135546	260.3686215	39,303.53325
Tablet #2	212.5455549	1.6013101	805	15.24184906	53,521.06714	63.14602973	239.5645392	44,790.42433

**Table 5:** Significance or non-significance of the difference in responses resulting from the release profile of different geometries using Tamhane post hoc test.

TAMHANE POST HOC		TO	FFBE	CR	SHC	STC	DC	EXDC	FF
95 rel	TO	-	✓	✓	✓	✓	✓	✓	✓
30 min		-	✓	✓	✓	✓	✓	✓	✓
AUC		-	✓	✓	✓	✓	✓	✓	✓
DE		-	-	✓	-	✓	✓	✓	✓
MDT		-	✓	✓	✓	✓	✓	✓	✓
VDT		-	✓	✓	✓	✓	✓	✓	✓
95 rel	FFBE		-	✓	✓	✓	✓	✓	✓
30 min			-	✓	-	✓	✓	✓	✓
AUC			-	✓	✓	✓	✓	✓	✓
DE			-	✓	-	-	✓	✓	✓
MDT			-	✓	-	✓	✓	✓	✓
VDT			-	✓	-	✓	✓	✓	✓
95 rel	CR			-	✓	✓	✓	✓	✓
30 min				-	✓	✓	✓	✓	✓
AUC				-	✓	✓	✓	✓	✓
DE				-	-	-	-	-	-
MDT				-	✓	✓	✓	✓	✓
VDT				-	✓	✓	✓	✓	✓
95 rel	SHC				-	-	✓	✓	-
30 min					-	-	✓	✓	-
AUC					-	-	✓	✓	-
DE					-	-	-	-	-
MDT					-	-	✓	✓	-
VDT					-	-	✓	✓	-
95 rel	STC					-	-	✓	✓
30 min						-	-	✓	-
AUC						-	-	✓	✓
DE						-	-	-	-
MDT						-	-	✓	-
VDT						-	-	✓	✓
95 rel	DC						-	-	✓
30 min							-	-	✓
AUC							-	-	✓
DE							-	-	✓
MDT							-	-	✓
VDT							-	-	✓
95 rel	EXDC							-	✓
30 min								-	✓
AUC								-	✓
DE								-	-
MDT								-	✓
VDT								-	✓
95 rel	FF								-
30 min									-
AUC									-
DE									-
MDT									-
VDT									-

## Data Availability

All data obtained during the study are available from the corresponding author on reasonable request.

## Appendix A

**Table A1:** Correlation of common feature variables with drug release profile response values.

		95 rel	30 min	AUC	DE	MDT	VDT	Mean
LWH	Pearson Correlation	−0.808	0.943	−0.808	0.805	−0.804	−0.724	0.815
	Sig (2-tailed)	0.000	0.000	0.000	0.000	0.000	0.000	
APV	Pearson Correlation	−0.625	0.769	−0.637	0.656	−0.598	−0.506	0.632
	Sig (2-tailed)	0.000	0.000	0.000	0.000	0.000	0.000	
H/L	Pearson Correlation	0.863	−0.760	0.846	−0.411	0.893	0.938	0.785
	Sig (2-tailed)	0.000	0.000	0.000	0.000	0.000	0.000	
S/V	Pearson Correlation	−0.898	0.987	−0.891	0.788	−0.908	−0.846	0.886
	Sig (2-tailed)	0.000	0.000	0.000	0.000	0.000	0.000	
Mean		0.799	0.865	0.796	0.665	0.801	0.754	

**Table A2:** General information of the one-way ANOVA test to check the significance of the difference in the mean release responses between the 8 tested geometries.

ANOVA					
		Sum of Squares	df	F	Sig.
95 rel (min)	Between Groups	48,224,641.000	7	153.651	0.000
	Within Groups	8,608,693.000	192		
	Total	56,833,334.000	199		
30 min (%)	Between Groups	1364.813	7	80.973	0.000
	Within Groups	462.312	192		
	Total	1827.125	199		
AUC (min)	Between Groups	200,697,225,162.955	7	176.070	0.000
	Within Groups	31,265,056,952.415	192		
	Total	231,962,282,115.370	199		
DE (%)	Between Groups	95.657	7	17.370	0.000
	Within Groups	151.053	192		
	Total	246.709	199		
MDT (min)	Between Groups	5,012,098.431	7	119.175	0.000
	Within Groups	1,153,550.106	192		
	Total	6,165,648.536	199		
VDT (min^2^)	Between Groups	1,528,939,045,215.330	7	95.221	0.000
	Within Groups	440,415,268,067.702	192		
	Total	1,969,354,313,283.030	199		

**Table A3:** Values of the parameters used in the simulations.

Study	De Piano et al. [40]			Lamberti et al. [25]	
Parameter	50 rpm	75 rpm	100 rpm		Description
*D1eq* (m^2^/s)	2 × 10^−10^ [40]	2 × 10^−10^ [40]	2 × 10^−10^ [40]	1 × 10^−10^	Diffusion coefficient of water in equilibrium state
*D2eq* (m^2^/s)	1.5 × 10^−10^ [40]	1.5 × 10^−10^ [40]	1.5 × 10^−10^ [40]	8 × 10^−11^	Diffusion coefficient of drug in equilibrium state
β_1_	5 [40]	5 [40]	5 [40]	5 [40]	Water beta constant
β_2_	2.5 [40]	2.5 [40]	2.5 [40]	2.5 [40]	Drug beta constant
*w*_10_	0 [40]	0 [40]	0 [40]	0.0217 [25]	Mass fraction of water at the initial moment
*w*_20_	0.5 [40]	0.5 [40]	0.5 [40]	0.4644 [25]	Mass fraction of drug at the initial moment
*w_1eq_*	0.97 [40]	0.97 [40]	0.97 [40]	0.97 [40]	Mass fraction of water in equilibrium state
*w_2eq_*	0 [40]	0 [40]	0 [40]	0 [40]	Mass fraction of drug in equilibrium state
*r*_1_ (kg/m^3^)	1000 [25]	1000 [25]	1000 [25]	1000 [25]	Water density
*r*_2_ (kg/m^3^)	1200 [25]	1200 [25]	1200 [25]	1200 [25]	Drug density
*r*_3_ (kg/m^3^)	1200 [25]	1200 [25]	1200 [25]	1200 [25]	Polymer density
*K_er_* (m/s)	1.3 × 10^−7^ [40]	1.8 × 10^−7^ [40]	2.2 × 10^−7^ [40]	6 × 10^−8^	Erosion constant
